# Deciphering the cellular and molecular landscapes of Wnt/β-catenin signaling in mouse embryonic kidney development

**DOI:** 10.1016/j.csbj.2024.08.025

**Published:** 2024-09-02

**Authors:** Hui Zhao, Hui Gong, Peide Zhu, Chang Sun, Wuping Sun, Yujin Zhou, Xiaoxiao Wu, Ailin Qiu, Xiaosha Wen, Jinde Zhang, Dixian Luo, Quan Liu, Yifan Li

**Affiliations:** a Department of Laboratory Medicine, Huazhong University of Science and Technology Union Shenzhen Hospital (Nanshan Hospital) and The 6th Affiliated Hospital of Shenzhen University Medical School, Shenzhen, Guangdong 518052, China; b Guangzhou National Laboratory, Guangzhou International Bio Island, No. 9 Xing Dao Huan Bei Road, Guangzhou 510005, Guangdong Province, China; c State Key Laboratory of Heavy Oil Processing, China University of Petroleum-Beijing, Beijing 102249, China; d College of Animal Science and Technology, Guangxi University, Nanning 530004, China; e Department of Pain Medicine, Shenzhen Municipal Key Laboratory for Pain Medicine, The affiliated Nanshan People's Hospital, The 6th Affiliated Hospital of Shenzhen University Medical School, Shenzhen 518060, China; f Institute of Pharmacy and Pharmacology, School of Pharmaceutical Science, Hengyang Medical School, University of South China, Hengyang, Hunan 421001, China; g Guangdong Medical University, Zhanjiang 524023, Guangdong China

**Keywords:** Wnt/β-catenin signaling, Embryonic kidney development, Gene expression profiling, Ureteric bud, Cap mesenchyme, Immune response in development

## Abstract

**Background:**

The Wnt/β-catenin signaling pathway is critical in kidney development, yet its specific effects on gene expression in different embryonic kidney cell types are not fully understood.

**Methods:**

Wnt/β-catenin signaling was activated in mouse E12.5 kidneys *in vitro* using CHIR99021, with RNA sequencing performed afterward, and the results were compared to DMSO controls (dataset GSE131240). Differential gene expression in ureteric buds and cap mesenchyme following pathway activation (datasets GSE20325 and GSE39583) was analyzed. Single-cell RNA-seq data from the Mouse Cell Atlas was used to link differentially expressed genes (DEGs) with kidney cell types. β-catenin ChIP-seq data (GSE39837) identified direct transcriptional targets.

**Results:**

Activation of Wnt/β-catenin signaling led to 917 significant DEGs, including the upregulation of *Notum* and *Apcdd1* and the downregulation of *Crym* and *Six2*. These DEGs were involved in kidney development and immune response. Single-cell analysis identified 787 DEGs across nineteen cell subtypes, with Macrophage_Apoe high cells showing the most pronounced enrichment of Wnt/β-catenin-activated genes. Gene expression profiles in ureteric buds and cap mesenchyme differed significantly upon β-catenin manipulation, with cap mesenchyme showing a unique set of DEGs. Analysis of β-catenin ChIP-seq data revealed 221 potential direct targets, including *Dpp6* and *Fgf12*.

**Conclusion:**

This study maps the complex gene expression driven by Wnt/β-catenin signaling in embryonic kidney cell types. The identified DEGs and β-catenin targets elucidate the molecular details of kidney development and the pathway's role in immune processes, providing a foundation for further research into Wnt/β-catenin signaling in kidney development and disease.

## Graphical Abstract

**Figure:**
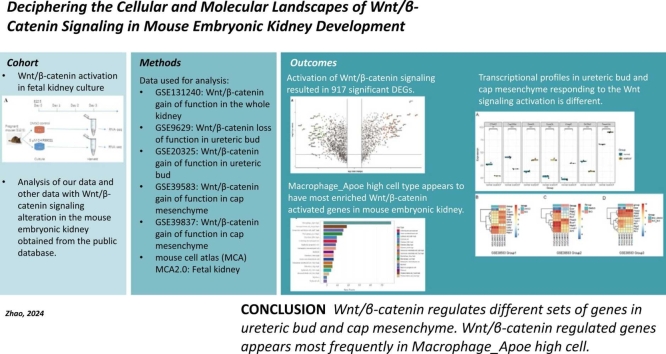

## 1 Introduction

Kidney development begins with reciprocal signaling between the ureteric bud, derived from the Wolffian duct, and the surrounding mesenchyme. This interaction leads to the sequential formation of the pronephros, mesonephros, and ultimately the metanephros, which becomes the adult kidney. During this process, the metanephric mesenchyme condenses into the cap mesenchyme, the precursor to the kidney's nephron progenitor. Nephron progenitors progress through stages like the renal vesicle and S-shaped bodies to form the nephron, comprising podocytes, tubules, and the loop of Henle. Simultaneously, the ureteric bud develops the urinary system, including the collecting ducts and ureter [1], [2]. Other cells, such as immune cells, especially macrophages [3], [4], [5], and endothelial cells, also contribute to this process. This developmental process, driven by ongoing signaling among the mesenchyme, ureteric bud, and stromal cells, continues until birth in humans and postnatally in mice [6].

The Wnt/β-catenin signaling pathway is essential for kidney development, orchestrating the complex morphogenetic events that shape the embryonic kidney [7]. Research indicates that the metanephric mesenchyme can be induced by Wnt1, Wnt3a, Wnt4, Wnt7a, and Wnt7b [8]. The most crucial Wnt molecules in kidney development are Wnt9b and Wnt4, which play roles in the early induction of cap mesenchyme cells into nephron units. Wnt4 is one of the main signals from the metanephric mesenchyme, and in Wnt4 knockout embryonic kidneys, pretubular cell aggregates do not form [9]. Wnt9b, secreted by the ureteric bud, is the primary signal molecule acting on the metanephric mesenchyme [10]. Wnt9b induces *Wnt4* expression in cap mesenchyme cells, and without Wnt9b, *Wnt4* is not expressed, leading to a lack of nephron formation and resulting in postnatal death in mice. Wnt9b-dependent expression of *Wnt4* starts in induced cap mesenchyme cells and continues until the S-shaped body stage, after which the expression ceases [9], [10], [11]. Besides its initial inductive role, Wnt9b is crucial for the convergent extension of renal tubule epithelial cells, while Wnt4 signaling also controls the fate of renal medullary smooth muscle cells. *Wnt11*, expressed in the ureteric bud, controls early ureteric bud branching and influences the final size of the kidney [12]. *Wnt11* knockout disrupts ureteric bud branching, causing renal dysplasia. *Wnt7b*, expressed in the ureteric bud, has specific functions in regulating medullary formation [13]. *Wnt7b* knockout prevents the formation of the medullary stripe and urine concentration [14].

The Wnt/β-catenin signaling pathway operates through the β-catenin molecule. It is clear that β-catenin expression and signaling are more pronounced in the ureteric bud and cap mesenchyme than in the developing stroma. In a previous study by the same group, β-catenin was predominantly found in the ureteric bud of the E18.5 rat kidney and was absent in the developing stroma [15]. This aligns with earlier studies that identified β-catenin and its signaling primarily in the ureteric bud [16], [17], [18]. Additional literature supports the intense localization of β-catenin in the branching ureteric bud [19], [20], [21], [22]. While immunohistochemistry results from the previous studies indicated β-catenin's main localization at the membrane/cytosol [15], nuclear staining of β-catenin in ureteric bud cells has been documented using immunofluorescence [18]. Active canonical WNT signaling, shown by TCF reporter activity, was evident in the branching ureteric bud as it transitions into renal tubules [19]. The expression of Axin2, a typical target of Wnt/β-catenin signaling, in the ureteric bud of the fetal kidney further supports the presence of this pathway [23]. Consistent with previous studies, the earlier study by the same group also revealed the presence of β-catenin in the cap mesenchyme [15], as shown by its expression in Six2-positive cap mesenchyme cells [24], [25], [26], Myc-positive nephron progenitor cells [27], and the nephrogenic mesenchyme [19], [20], [21], [22]. Additionally, active canonical WNT signaling was observed in the epithelia of the nephrogenic mesenchyme [19]. However, some studies report an absence of β-catenin activity in nephron progenitors [28], [29], which may be due to the lack of high-resolution images necessary to detect β-catenin staining and TCF reporter activity in the cap mesenchyme. Previous studies by the same group also confirm that β-catenin is not present in developing podocytes [15], consistent with findings that TCF reporter activity is downregulated in maturing nephrons and becomes undetectable postnatally [19]. Furthermore, the previous study by the same group supports the notion that β-catenin is not expressed in the developing stroma, which is consistent with most of the literature. However, some studies have detected β-catenin in the developing stroma [14], [30] and the medullary stroma of the developing human kidney [20]. This inconsistency may be due to the varying specificity and sensitivity of β-catenin antibodies and the detection methods used (IHC vs. immunofluorescence). While conditional deletion of β-catenin in the stroma, driven by the *Foxd1* promoter, has been shown to affect kidney development [14], [30], the majority of evidence suggests that β-catenin is not expressed in the developing stroma [16], [17], [18], [19], [21], [22], [24], [25], [26], [27]. The role of Wnt/β-catenin signaling in the stroma also requires further validation through results from TCF luciferase reporter activity [14], [30].

While the presence and localization of β-catenin in the ureteric bud and cap mesenchyme of the developing kidney have been well-documented, the broader landscape of Wnt/β-catenin signaling intensity and its target genes across various embryonic kidney tissues remains largely unexplored. This study aims to unravel the complexities of Wnt/β-catenin signaling within different kidney tissues by leveraging high-throughput RNA sequencing, microarray, and single-cell analysis. By activating the Wnt/β-catenin pathway *in vitro* in mouse E12.5 kidneys and employing comprehensive bioinformatic analyses, this study seeks to delineate differential gene expression profiles and identify potential direct targets of β-catenin within the whole embryonic kidney. The approach also includes examining publicly available datasets to contrast gene expression profiles associated with activated versus inhibited Wnt/β-catenin signaling, particularly focusing on the ureteric bud—a region where β-catenin signaling is known to be prominent—as well as the cap mesenchyme.

Furthermore, the study delves into the single-cell transcriptional landscape of the embryonic kidney to understand the heterogeneity and cell-type specificity of Wnt/β-catenin signaling. By mapping differentially expressed genes to canonical cell-type markers, this study aims to uncover the nuances of pathway activation across diverse kidney cell populations. The integration of ChIP-seq data allows the identification of genes that could be directly regulated by β-catenin, offering a more detailed view of its transcriptional influence. Collectively, the findings of this study are poised to provide a refined understanding of the Wnt/β-catenin signaling network during kidney development, highlighting its variable impact across different cellular contexts and contributing to the broader comprehension of renal morphogenesis and pathology.

The innovative aspect of our study lies in the comprehensive differential gene expression profiling induced by the activation and modulation of the Wnt/β-catenin signaling pathway in embryonic mouse kidneys. By using CHIR99021 treatment and leveraging RNA sequencing data, our research identified a significant number of differentially expressed genes (DEGs) with profound implications for renal development and immune response. We mapped these DEGs to the Gene Ontology (GO) and KEGG databases, uncovering their involvement in crucial developmental pathways and immune responses, which have not been extensively characterized before. By focusing on the distinct transcriptomic changes in the ureteric bud and cap mesenchyme upon Wnt signaling activation and integrating β-catenin ChIP-seq data to identify direct targets of β-catenin, our study provides new insights into the specific roles of β-catenin in kidney development at a cellular level. The single-cell RNA-seq analysis further highlights the cellular diversity affected by Wnt/β-catenin signaling, especially its effect on the Apoe-high macrophages, pinpointing key cell types and genes involved in this process.

## 2 Materials and Methods

### 2.1 Sample Collection

Murine embryonic kidney tissues (C57BL/6) were sourced from the Guangdong Medical Laboratory Animal Center, with experiments conducted in accordance with the Ministry of Science and Technology of China's animal care guidelines (2006) and approved by the ethics committee of Huazhong University of Science and Technology Union Shenzhen Hospital. Eight tissues were analyzed, with the sample size determined by ethical considerations, standard practices in the field, replication for reliability, and practical constraints. To assess Wnt/β-catenin signaling on gene expression in renal development, mRNA profiles from E12.5 mouse embryonic kidneys cultured *in vitro* and treated with CHIR99021 (5 µM) or DMSO (0.1 %) for 72 hours were deep-sequenced in quadruplicate (Supplementary Figure 4). CHIR99021 is a highly selective GSK-3β inhibitor that can activate Wnt/β-catenin signaling. Multiple studies use CHIR99021 at the micromolar (µM) level in their experiments with fetal kidney cells or kidney organoids generated from induced pluripotent stem cells (iPSCs) or embryonic stem (ES) cells. For example, CHIR99021 at 3 µM was used to treat embryonic day (E) 14–15.5 kidney cells [31]. CHIR99021 at 8 µM and 5 µM has been used for the generation of kidney organoids and regulation of nephrogenesis in these organoids [32], [33]. Another recent paper used CHIR99021 at different concentrations (1 µM, 3 µM, 5 µM, 10 µM), ranging from 1–10 µM [34]. The methodology for kidney organ culture was as previously described [35], and data were submitted to NCBI GEO under accession No. GSE131240.

For the RNA-seq experiment, this study implemented predefined criteria to select C57BL/6 mice, ensuring genetic uniformity. Pregnant mice at 11 weeks were included to reduce age-related gene expression variability, and only those passing veterinary inspection without signs of infection or disease were selected. Exclusion criteria included animals with health issues or distress during pregnancy and embryonic kidneys unresponsive to treatment, as indicated by typical Wnt/β-catenin target gene changes. To control for variability, the study employed a paired design, allocating the left kidney of each embryo to the control group and the right kidney to the treatment group, thus avoiding traditional randomization. This matched pairs design inherently adjusts for genetic and environmental differences across subjects, aiming to mitigate biases from individual embryonic variability. Uniform environmental conditions were maintained in the *in vitro* kidney organ culture experiment by using a single CO^2^ incubator and standardizing the spatial positioning of all petri dishes on the same incubator shelf.

### 2.2 High-throughput RNA-seq and data analysis

Total RNA was extracted from each sample using the EASYspin Plus kit (Aidlab Biotech, RN2802) and assessed for purity, concentration, and integrity using NanoPhotometer® spectrophotometry (IMPLEN, CA, USA), Qubit® 3.0 Fluorometry (Life Technologies, CA, USA), and Agilent 2100 RNA Nano 6000 Assay Kit (Agilent Technologies, CA, USA), respectively. The RNA libraries for sequencing were prepared with the Hieff NGS® MaxUp™ II Kit (Cat#12301ES96) and sequenced on the Illumina Novaseq platform (IlluminaInc., CA, USA), yielding an average of 42.60 million paired-end reads per sample. Reads were filtered to remove adaptor contamination, low-quality sequences, and those with excessive "N" bases. Clean reads were aligned to the mouse GRCm38-mm10 genome using HISAT2 and quantified by featureCounts, with expression levels normalized to FPKM. Differential expression analysis was conducted using the R package ‘limma’ (Version 3.56.2). Further details on DEGs analysis are provided in the statistical and functional analysis sections. The RNA extraction and mRNA sequencing were performed at Annoroad Company (Beijing, China).

### 2.3 Study datasets

Four RNA microarray datasets (GSE39583 [36], GSE9629 [16], GSE20325 [37]) and one ChIP-seq dataset (GSE39837 [36]) were acquired from the Gene Expression Omnibus (GEO), along with the RNA-seq dataset from this study (GSE131240) (Supplementary Table 8). The selection criteria for these datasets included comprehensive platform documentation for analytical reliability and relevance to altered Wnt/β-catenin signaling in fetal mouse kidneys.

Dataset GSE9629 characterizes β-catenin loss of function in the ureteric bud of E12.5 mice with conditional β-catenin alleles disrupted by LoxP sites flanking exons 2 through 6 [16], bred with Hoxb7- Cre:GFP mice. Meanwhile, GSE20325 contains data from mice with a targeted gain-of-function mutation in the β-catenin gene (β-catdelta3/delta3), achieved by breeding Hoxb7-Cre mice with those carrying loxP-flanked exon 3, affecting the ureteric bud [37].

The GSE39583 dataset details the transcriptional response of cap mesenchyme, defined as Six2-positive nephron progenitors from E16.5 mice, to Wnt stimulation via 4 μM BIO treatment for 24 and 48 hours (Supplementary Figure 3) [26]. Dataset GSE39837 comprises ChIP-seq data from Six2-positive cells at E16.5, which were FACS-sorted, pelleted by centrifugation, and cultured in DMEM with 10 % FBS and 4 μM BIO for 24 hours for β-catenin ChIP analysis [26].

The study combined fetal kidney cell subtype information from the Mouse Cell Atlas (MCA) version 2.0 (https://bis.zju.edu.cn/MCA/gallery.html?tissue=Fetal-Kidney) with differentially expressed genes (DEGs) from this study (GSE131240) to categorize DEGs by cell subtype, detailed in Supplementary Table 8. The MCA database encompasses over ten mouse tissues across various developmental stages [38], [39], [40]. Single-cell RNA-seq data of 9432 fetal kidney cells at E10.5, E12.5, and E14.5 were sourced to align with RNA-seq data from this study [39].

### 2.4 Statistical analysis

Statistical analysis was conducted using R software (Version 4.0.2), employing the 'limma' package (Version 3.56.2) to identify DEGs across experimental and control groups within various gene expression datasets. The process began with a Log2 transformation of the expression matrix to approximate a normal distribution, followed by inter-array normalization using 'normalizeBetweenArrays' to reduce technical variance. DEG identification was performed through linear modeling with weighted least squares in 'limma', complemented by Bayesian methods for multiple testing correction. Models were fitted using 'lmFit', and 'eBayes' was applied to leverage Bayesian statistics for DEG detection.

### 2.5 Functional analyses of DEGs

Functional characterization of DEGs was performed using Gene Ontology (GO) and Kyoto Encyclopedia of Genes and Genomes (KEGG) pathway enrichment analyses utilizing the "enrichGO" and "enrichKEGG" functions from the R package clusterProfiler (Version 4.4.4). Associations between genes and GO terms were visualized using "cnetplot". The "tidyr" package (Version 3.1.0) facilitated the segregation of gene and background ratios into GR1, GR2, and BR1, BR2, respectively. The enrichment factor for GO terms was determined using the formula: enrichment factor = (as.numeric(GR1) / as.numeric(GR2)) / (as.numeric(BR1) / as.numeric(BR2)), indicating the direction of GO term enrichment.

### 2.6 Availability of data

The sequencing data for the eight murine embryo kidney samples were deposited in the NCBI with the accession number GSE131240.

## 3 Results

### 3.1 Differential gene expression profiling of activated Wnt/β-catenin signaling in the whole embryonic mouse kidney

**Fig. 1:**
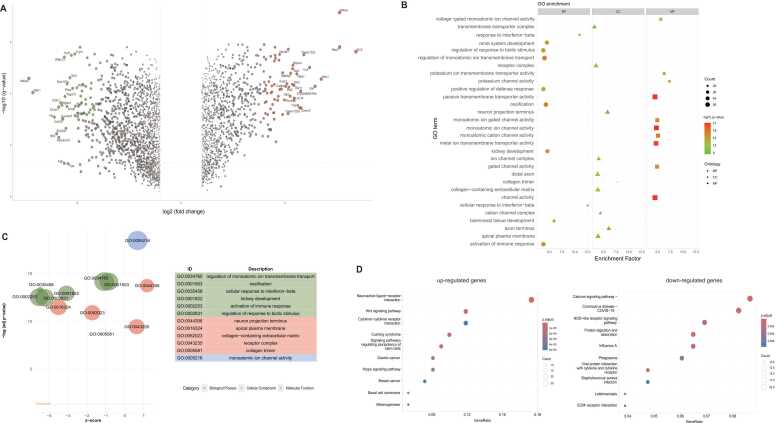
**The DEGs and function analysis. (A)** Volcano plot of the DEGs. DEGs were derived by analysis of the RNA-seq data GSE131240; each point represents one gene, genes shown in the red color were selected by adjusted p-value < 1e-3 and Log FC > 4 (case/control); the genes in green color represent the adjusted p-value < 1e-3 and Log FC < −4. **(B)** The GO analysis of the DEGs. The significantly differentially expressed gene list was determined by the adjusted p-value < 1e-2 and |Log FC= > 2 condition. The x-axis was the enrichment factor (for detailed calculation, please refer to the Method section). The y-axis was the GO term. This figure is divided into three parts, each representing one of the three major semantic categories of GO terms, including Biological Process / Cellular Component / Molecular Function. **(C)** The GO bubble plot. The x-axis was the z-score. The positive value represents the GO term is more likely to be increased, while the negative represents the GO term to be decreased. **(D)** The KEGG pathway analysis. The left figure showed the upregulated genes enriched pathways and the right figure showed the downregulated genes enriched KEGG pathways.

Mouse kidneys at embryonic day 12.5 (E12.5) were treated with CHIR99021 for three days *in vitro* to activate Wnt/β-catenin signaling, with DMSO-treated wild-type kidneys serving as controls. Four replicates per treatment were analyzed via RNA sequencing (GSE131240), identifying 917 significant DEGs (|Log FC= > 1, FDR < 0.05, Supplementary Table 1). Notably, genes such as palmitoleoyl-protein carboxylesterase (*Notum*), transmembrane protein 132 C (*Tmem132c*), neurotensin receptor 2 (*Ntsr2*), engrailed homeobox 2 (*EN2*), and APC down-regulated 1 (*Apcdd1*) were significantly upregulated, while crystallin, mu (*Crym*), TNF receptor-associated factor 1 (*Traf1*), WAP four-disulfide core domain 13 (*Wfdc13*), SIX homeobox 2 (*Six2*), neurotensin receptor 1 (*Ntsr1*), and mesenchyme homeobox 2 (*Meox2*) were notably downregulated in Wnt/β-catenin activated kidneys (FDR < 1e-3, |Log(FC)| > 4) (Fig. 1A).

This study explored the roles of DEGs by mapping them to the Gene Ontology (GO) database, revealing enrichment in system development pathways, notably renal system and kidney development (FDR < 1e-2, |LogFC= > 2) (Fig. 1B). The DEGs were also linked to immunological processes, including the activation of the immune response, positive regulation of defense response, and response to interferon-beta (FDR < 1e-2, |LogFC= > 2) (Fig. 1B). Specific GO terms such as regulation of monoatomic ion transmembrane transport, ossification, cellular response to interferon-beta, kidney development, activation of immune response, regulation of response to biotic stimulus, apical plasma membrane, collagen-containing extracellular matrix, and collagen trimer were downregulated. In contrast, receptor complex, neuron projection terminus, and monoatomic ion channel activity were upregulated (Fig. 1C). Furthermore, the study identified genes associated with these pathways, including DEP domain containing 1a (*Depdc1a*) and transferrin receptor (*Tfrc*) with E2F target processes, and C-X3-C motif chemokine ligand 1 (*Cx3cl1*) and Kruppel-like transcription factor 6 (*Klf6*) with the inflammatory response (Supplementary Figure 1).

In this study, KEGG analysis was conducted to determine the pathways of the upregulated and downregulated genes. It was found that 334 upregulated genes were enriched in the Wnt signaling pathway, neuroactive ligand-receptor interaction pathway, cytokine-cytokine receptor interaction pathway, signaling pathways regulating pluripotency of stem cells, and Hippo signaling pathway (Fig. 1D). Additionally, 468 downregulated genes were enriched in the calcium signaling pathway, COVID-19 pathway, NOD-like receptor signaling pathway, and protein digestion and absorption (Fig. 1D).

### 3.2 Differential gene expression profiling of altered Wnt/β-catenin signaling in mouse embryonic kidney ureteric buds

**Fig. 2:**
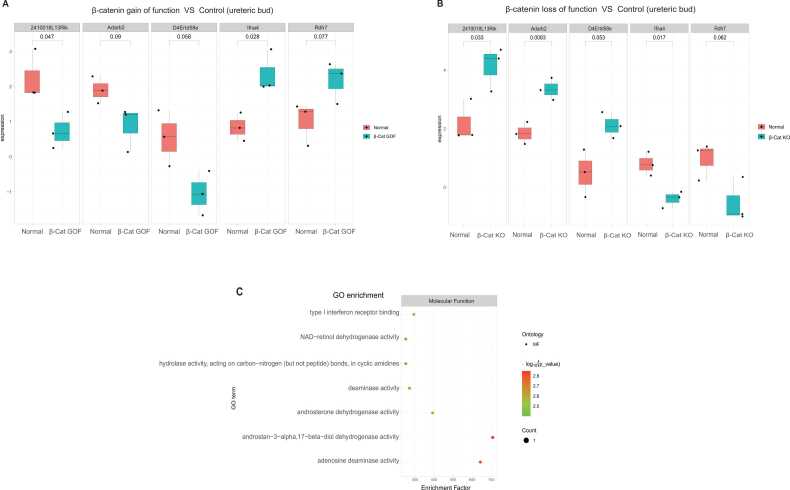
**The gene expression profile of the β-catenin deficient and activated dataset. (A)** Boxplot of the DEGs for dataset GSE20325 (gain of function in ureteric bud). The DEGs were selected by the following conditions: p-value < 5e-2 and |Log(FC)| > 1 (case/control). **(B)** The boxplot of the DEGs for dataset GSE9629 (loss of function in ureteric bud). The DEGs were selected by the following conditions: p-value < 5e-2 and |Log(FC)| > 1. **(C)** GO term analysis of the significantly differentially expressed genes, the x-axis was the enrichment factor.

Previous studies have reported that β-catenin is mainly expressed in ureteric buds [16], [17], [18]. Leveraging datasets GSE20325 (β-catenin gain of function in ureteric bud, Supplementary Table 2) and GSE9629 (β-catenin loss of function in ureteric bud, Supplementary Table 3), this study investigated the impact of β-catenin modulation on gene expression in ureteric buds. This analysis sought genes with inverse expression patterns between β-catenin activation (gain of function) and inhibition (loss of function). Five genes with such differential expression were identified. *Rdh7* and *Ifna4* were upregulated with β-catenin activation but downregulated upon its deficiency, while *Adarb2*, *D4Ertd58e*, and *2410018L13Rik* showed the opposite trend (p-value < 5e-2, |Log FC= >1) (Figs. 2A, 2B).

GO analysis on these five genes revealed enrichment in molecular function GO terms, such as type I interferon receptor binding, NAD-retinol dehydrogenase activity, androsterone dehydrogenase activity, and deaminase activity (Fig. 2C). The consistent inverse expression patterns of these five genes following the activation or inhibition of β-catenin reliably establish the regulatory impact of the transcription factor β-catenin on these genes (Figs. 2A, 2B).

### 3.3 The different transcript profiles in ureteric bud and cap mesenchyme after activation of the Wnt signaling pathway

**Fig. 3:**
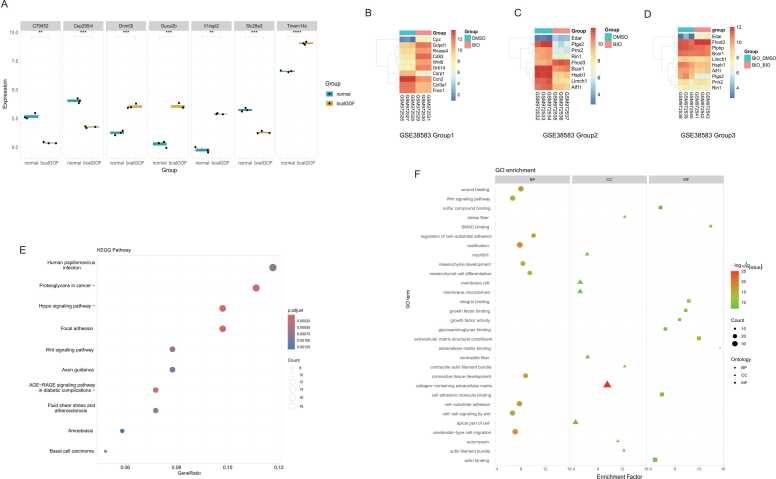
**Transcriptional response of ureteric bud and cap mesenchyme to Wnt activation. (A)** Boxplot of the DEGs for the dataset GSE20325. DEGs were selected by the following conditions: adjusted p-value < 5e-2 and |Log(FC)| > 1 (case/control). **(B-D)** Heatmap of the DEGs of GSE39583. DEGs were selected by the following conditions: adjusted p-value < 5e-2 and |Log(FC)| > 1. **(E)** KEGG pathway analysis for the 294 significant expressed genes in cap mesenchyme. The x-axis shows the gene ratio. **(F)** GO term analysis of the significantly expressed genes in cap mesenchyme after the Wnt pathway activation. The x-axis represents the enrichment factor.

Transcriptomic changes in the ureteric bud and cap mesenchyme in response to Wnt signaling activation were analyzed using datasets GSE20325 (β-catenin gain of function in the ureteric bud) [37] and GSE39583 (β-catenin gain of function in the cap mesenchyme). In the ureteric bud, seven genes showed significant differential expression upon Wnt pathway activation: *C79452, Cep295nl,* and *Slc28a3* were downregulated; *Dnmt3l*, *Guca2b*, *Il1rapl2*, and *Tmem14c* were upregulated (FDR < 5e-2, |Log FC= >1) (Fig. 3A). In the cap mesenchyme, 294 genes exhibited significant differential expression across three experimental groups treated with BIO versus DMSO controls (FDR < 5e-2 and |Log FC= > 1) (Supplementary Tables 4 and 5). Notably, genes like *Cpz, Gdpd1, Rnase4, Cd83, Wnt6,* and *Grb14* were upregulated in the first group, while *Csrp1, Ccn2*, *Col3a1*, and *Foxc1* were downregulated (Fig. 3B). The second group showed general downregulation of genes such as *Bcar1* and *Limch1*, with *Fhod3* and *Edar* upregulated (Fig. 3C). Similarly, group three saw upregulation of *Edar* and downregulation of genes including *Ptgs2* and *Hspb1* (Fig. 3D).

Due to the paucity of significantly differentially expressed genes in GSE20325, functional enrichment analysis was not performed. However, analysis of the 294 DEGs in the cap mesenchyme revealed substantial enrichment in KEGG pathways, including Wnt signaling, Hippo signaling, focal adhesion, and AGE-RAGE signaling in diabetic complications (Fig. 3E). GO term analysis indicated these DEGs were predominantly associated with the Wnt signaling pathway, regulation of cell-substrate adhesion, mesenchyme development, and cell−cell signaling by the Wnt biological process (Fig. 3F), with a general trend of decrease (Supplementary Figure 2). These findings suggest distinct downstream signaling effects following Wnt pathway activation in the ureteric bud and cap mesenchyme.

### 3.4 Identification of target gene based on the β-catenin ChIP-Seq data

**Fig. 4:**
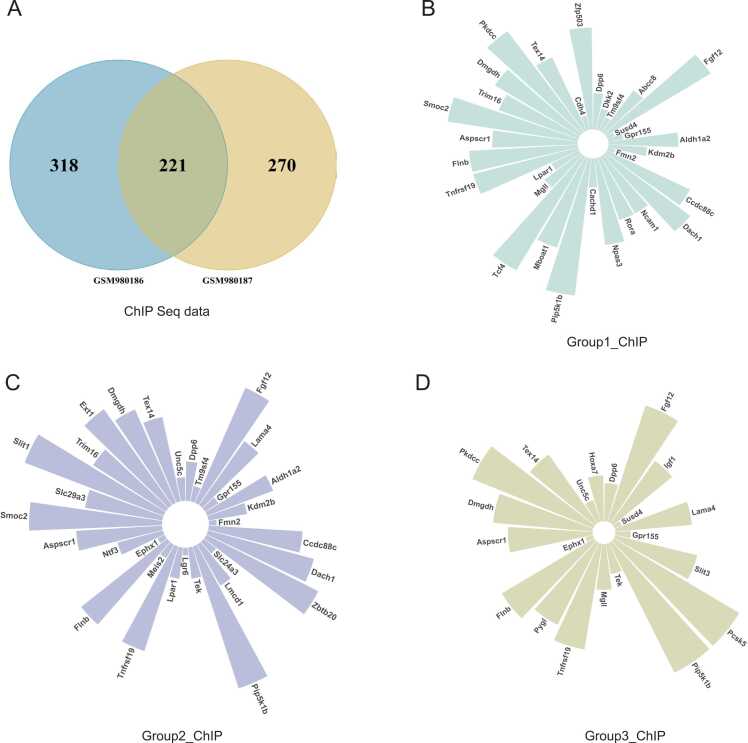
**The ChIP-Seq analysis of the β-catenin ChIP dataset GSE39837. (A)** For the two biological replicate samples for the β-catenin ChIP data, the peak numbers for each biological replicate were shown in the figure. **(B-D)** The overlap genes of the β-catenin ChIP data and β-catenin gain of function DEGs in cap mesenchyme for group 1 (B), group 2 (C), and group 3 (D) of dataset GSE39583. The length of each circle barplot was the chromosome ID. The gene in each chromosome was texted in the figure.

To identify genes directly regulated by β-catenin, this study integrated a β-catenin ChIP-seq dataset (GSE39837) with a gene expression dataset (GSE39583), mapping ChIP peaks to gene loci (Supplementary Table 6). 221 genes were identified as potential direct targets of β-catenin, including notable genes such as *Accn1* and *Stra13* on chromosome 11, *Fam19a5* on chromosome 15, *Dpp6* and *A930011G23Rik* on chromosome 5, and *Dkk2* and *Mecom* on chromosome 3 (Fig. 4A, Supplementary Table 6). The study identified 31, 31, and 21 genes overlapping between the β-catenin ChIP-seq data (GSE39837) and groups 1, 2, and 3 from the β-catenin activation data in cap mesenchyme (GSE39583) (Figs. 4B, 4C, 4D). In group 1, genes such as *Dpp6*, *Tm9sf4*, *Tnfrsf19*, and *Fgf12* were identified as directly regulated by β-catenin (Fig. 4B). In group 2, *Dpp6*, *Tm9sf4*, *Fgf12*, and *Tnfrsf19* were found to be target genes of β-catenin (Fig. 4C). In group 3, *Dpp6*, *Fgf12*, and *Tnfrsf19* were also identified as directly impacted by β-catenin (Fig. 4D). The direct regulatory effect of β-catenin on these target genes was present in at least two groups.

### 3.5 Single-cell analysis of mouse kidney

**Fig. 5:**
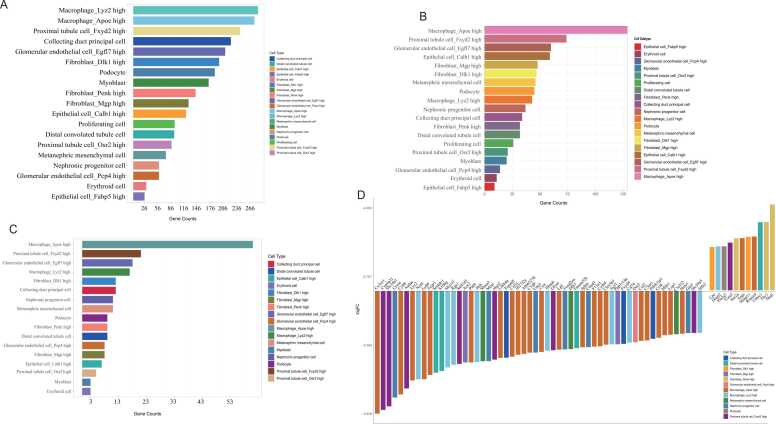
**Cell types of differentially expressed genes in GSE131240.** (A) Number of marker genes for 19 fetal kidney cell types in the MCA database. (B) Gene counts of the overlap of DEGs in GSE131240 (FDR < 0.05) and the cell marker genes from the MCA database, grouped by cell types. (C) Gene counts of the overlap of DEGs in GSE131240 (FDR < 0.05, |Log(FC)| > 1) and the cell marker genes from the MCA database, grouped by cell types. (D) The fold change of the top 67 DEGs in GSE131240 (FDR < 0.05, |Log(FC)| > 1) that are overlap with the cell marker genes from the MCA database, grouped by cell types. Supplementary Figure 1. The correlation of the GO Terms and genes for the dataset GSE131240. The figure shows the genes correlated with GO terms, the circle in the middle represents the GO term, and genes are connected by a line segment. Supplementary Figure 2. The GO bubble plot for the 294 significantly differentially expressed genes in cap mesenchyme (dataset GSE39583). The x-axis was the z-score. The positive value represents the GO term, which is more likely to be increased, while the negative represents the GO term, which is likely to be decreased. Supplementary Figure 3. The experimental design involved treating cap mesenchyme cells, which are undifferentiated nephron progenitors, with 4 μM BIO to activate Wnt signaling, as documented in dataset GSE39583.

Cell-type canonical marker genes from mouse single-cell data were derived from the publicly released single-cell RNA-seq dataset in the Mouse Cell Atlas (MCA) database. The canonical marker gene count of Macrophage_Lyz2 high ranked the highest, followed by Macrophage_Apoe high, Proximal tubule cell_Fxyd2 high, Collecting duct principal cell, Glomerular endothelial cell_Egfl7 high, and Fibroblast_Dlk1 high. The differences in gene counts among these different cell types were small (Fig. 5A).

To elucidate the cellular diversity influenced by Wnt/β-Catenin signaling in embryonic kidney development, differentially expressed genes (DEGs, FDR < 0.05) from dataset GSE131240 were mapped against 2718 canonical cell-type markers of the embryonic kidney from the Mouse Cell Atlas. This analysis identified 787 genes shared between these datasets with significant adjusted p-values (adjusted p-value < 0.05) (Supplementary Table 7). These genes were distributed across nineteen cell subtypes (Fig. 5B), with the highest gene counts in Macrophage_Apoe high, followed by Proximal tubule cell_Fxyd2 high, Glomerular endothelial cell_Egfl7 high, Epithelial cell_Calb1 high, Fibroblast_Mgp high, and Fibroblast_Dlk1 high. Notably, Macrophage_Apoe had gene counts more than double that of other cell types (Fig. 5B).

Next, dataset GSE131240 was sub-grouped by mapping the DEGs (FDR < 0.05, |Log(FC)| > 1) to cell-type canonical marker genes of mouse single-cell data from the Mouse Cell Atlas. Macrophage_Apoe high exhibited the highest gene counts, surpassing those in Proximal tubule cell_Fxyd2 high, Glomerular endothelial cell_Egfl7 high, Macrophage_Lyz2 high, and Fibroblast_Dlk1 high (Fig. 5C). Notably, the gene counts in Macrophage_Apoe high were more than threefold higher than those in other cell types (Fig. 5C).

The genes with more than a four-fold change were displayed (Fig. 5D). In Macrophage_Apoe high cells, 23 genes (*Cx3cr1, Cybb, Lyz2, Ly86, Fcgr1, Pld4, Lilrb4a, Csf1r, Ifi27l2a, AI662270, C1qb, Ctss, Lpl, Ighm, F13a1, Cyth4, Clec4a1, Ccl2, C3ar1, Ccl6, Milr1, Lcp1* and *Hcls1*) were downregulated, while *Srgn* and *Rnase4* were upregulated (Fig. 5B). In Macrophage_Lyz2 high cells, *Ncf4*, *Myo1f*, *Rgs1*, *Hck*, *Ifitm6*, *Ncf1*, *Cd74*, and *Slfn2* were downregulated, and *Dusp2* was upregulated. In Proximal tubule cell_Fxyd2 high cells, *Cyp4a31, Slc34a1, Aadac, Sult1d1, Hao2, Glyat* and *Slc39a5* were downregulated, while *Tcea3* was upregulated. In Fibroblast_Mgp high cells, *Ace2* and *Ppp1r14a* were downregulated. In Fibroblast_Dlk1 high cells, *Cpe* and *Mmp11* were upregulated. In Fibroblast_Penk high cells, *Itm2a*, *Thy1*, and *Nkd1* were upregulated. In nephron progenitor cells, *Crym*, *Mest*, *Uncx*, *Foxd2os*, *Cited1*, and *Gas1* were downregulated. In metanephric mesenchymal cells, *Shisa3*, *Dlk1*, and *Cxcl12* were downregulated. In podocytes, *Pla2g7* was upregulated. In Glomerular endothelial cell_Pcp4 high cells, *Osr2* was downregulated. In collecting duct principal cells, *Tspan8*, *Fxyd4*, and *Pdzk1ip1* were downregulated. In distal convoluted tubule cells, *Sostdc1*, *S100g*, and *Tmem52b* were downregulated, while *Cthrc1* was upregulated (Fig. 5B). Collectively, these data suggest that β-catenin activation regulates specific DEGs across diverse cell types, with Macrophage_Apoe high cells showing the most pronounced enrichment of Wnt/β-catenin-activated genes.

## 4 Discussion

This study significantly advances the understanding of the Wnt/β-catenin signaling pathway's role in embryonic kidney development. The identification of 917 DEGs upon Wnt activation highlights the pathway's broad regulatory influence, affecting genes involved in renal system development and immune responses. Noteworthy upregulated genes like *Notum* and *EN2*, and downregulated genes like *Six2* and *Traf1* (Fig. 1A), suggest potential targets for further investigation. The study compares gene expression profiles associated with activated versus inhibited Wnt/β-catenin signaling, particularly focusing on the ureteric bud and cap mesenchyme. This comparative analysis provides insights into the context-dependent regulation of gene expression by β-catenin, which is crucial for understanding its diverse functions in kidney development. The single-cell RNA-seq analysis highlights the cellular diversity influenced by Wnt/β-catenin signaling, particularly its impact on macrophages with high Apoe expression.

β-catenin 's alteration led to gene differential expression in the ureteric bud (e.g., *Rdh7*, *Ifna4*) (Figs. 2A, 2B) and cap mesenchyme (e.g., *Csrp1*, *Cpz*) (Fig. 3B), with significant changes in pathways like Wnt and hippo signaling (Fig. 3E). ChIP-Seq and gene expression data revealed potential β-catenin targets (e.g., *Dpp6*, *Dkk2*, *Fgf12*) in the cap mesenchyme (Fig. 4B). Single-cell analysis showed cell-specific responses to Wnt/β-catenin signaling in the kidney, notably affecting genes in nephrogenic progenitor cells (Fig. 5B). These insights into β-catenin's role in kidney development offer a detailed view of its regulatory networks and may inform renal pathology research.

In the GSE131240 dataset (Gain of function in the whole kidney), genes including *Notum*, *Tmem132c*, *Ntsr2*, *EN2*, and *Apcdd1* were upregulated in Wnt/β-catenin-activated kidney tissues (Fig. 1A), hinting at their roles in Wnt/β-catenin signaling effects in kidney development. *Notum*, a Wnt pathway inhibitor [41], and *Apcdd1*, a Wnt inhibitor and target [42], [43], may reflect a feedback mechanism in response to Wnt/β-catenin signaling. A previous study shows renal agenesis in some *Notum* (-/-) mice [44], indicating that *NOTUM* has a role in kidney development. *TMEM132C* is a member of the TMEM132 family, known to interact with the Wnt signaling pathway. *TMEM132A*, another member of this family, has been identified as a regulator of Wnt signaling through its interaction with Wntless (WLS) [45]. Neurotensin exerts its effects primarily through two receptor subtypes, NTSR1 and NTSR2. In glioblastoma cells, *NTSR1* expression is increased by the Wnt pathway activator Wnt3a and decreased by the Wnt inhibitor iCRT3 [46]. *EN2* expression is also Wnt-activated [47]. This study provides the first evidence that targets such as *Tmem132c* and *Ntsr2* could be upregulated by Wnt/β-catenin signaling in the context of kidney development. Further knockout studies to explore their roles in kidney development are essential, as demonstrated by the insights gained from the *Notum* knockout study [44].

The downregulation of genes such as *Ntsr1*, *Wfdc13*, *Traf1*, *Crym*, *Six2*, and *Meox2* in response to Wnt/β-catenin activation suggests that they may be suppressed by this pathway (Fig. 1A). *Meox2* is a developmental homeobox protein and potential tumor suppressor identified in Wilms tumor [48], arising from the developing kidney. *Ntsr1* has been reported to be upregulated by Wnt in glioblastoma [46], which contrasts with its downregulation in the fetal kidney shown in this study, indicating context-dependent regulation. Wnt also downregulates *Six2*, consistent with findings in human renal progenitor cells [49]. Notably, Wnt activation results in opposing regulation of *Ntsr1* and *Ntsr2* (Fig. 1A). *Ntsr1* was downregulated by Wnt/β-catenin activation, contrast to previous report showing that *NTSR1* expression is increased by the Wnt pathway activator Wnt3a [46], suggesting a context dependent regulation. The downregulation of *Meox2*, *Wfdc13*, *Traf1*, and *Crym* has not been previously reported, offering new insights into Wnt/β-catenin's role in kidney function or disease, warranting further investigation.

The link between DEGs and immune processes, such as immune activation, interferon-beta response (Figs. 1B, 1C) and cytokine-cytokine receptor interaction pathways (Fig. 1D), suggests that Wnt/β-catenin signaling may play a dual role in kidney development and immune regulation within the organ. This concept is supported by studies indicating the importance of immune components in the developing kidney [3], [4], other organ development [50], [51], and the involvement of aberrant Wnt signaling in chronic inflammation [52]. The reduced expression of immune-related genes could represent a mechanism to limit inflammation during kidney development, protecting against damage and promoting proper organ formation. Additionally, immune activity in the embryonic kidney could be part of the immune system's maturation, as shown by single-cell sequencing studies [53].

The upregulation of genes associated with neuron projection termini (Figs. 1B, 1C) and neuroactive ligand-receptor (Fig. 1D) suggests an interplay between Wnt/β-catenin signaling and neural development or signaling within the kidney. This supports the idea that the kidney has a sensory role, responding to both internal and external stimuli [54], [55], [56], and that Wnt/β-catenin signaling could contribute to the development of this function.

The upregulation of Wnt signaling suggest the Wnt/β-catenin signaling has been activated by the CHIR99021 treatment (Fig. 1D). The upregulation of genes in the Hippo signaling pathway (Fig. 1D), key for cell proliferation and differentiation, highlights its role in kidney development. This is consistent with previous report showing crosstalk between Wnt and Hippo signaling [57]. Conversely, the downregulation of genes in the calcium and NOD-like receptor signaling pathways (Fig. 1D), which are vital for inflammation and immune responses [58], [59], may indicate a developmental stage-specific attenuation of these pathways to support kidney development.

This study shows for the first time that *Rdh7* and *Ifna4* were upregulated in β-catenin deficient kidneys, while *Adarb2*, *D4Ertd58e*, and *2410018L13Rik* were downregulated in β-catenin gain-of-function ureteric buds (Figs. 2A, 2B). Gene Ontology analysis showed these genes are associated with functions such as type I interferon receptor binding and various dehydrogenase and deaminase activities (Fig. 2C), suggesting that β-catenin may affect kidney development through these molecular functions.

This study investigated the transcriptional profiles of the ureteric bud and cap mesenchyme in response to Wnt/β-catenin signaling pathway activation. Analysis of the datasets GSE20325 (β-catenin gain of function in ureteric bud) and GSE39583 (β-catenin gain of function in cap mesenchyme) revealed differential gene expression patterns in these two structures following Wnt/β-catenin pathway activation.

Significant transcriptional alterations were detected in the ureteric bud, with seven genes notably affected: three were downregulated (*C79452, Cep295nl, Slc28a3*), suggesting a potential inhibitory effect by Wnt/β-catenin signaling, and four were upregulated (*Dnmt3l, Guca2b, Il1rapl2, Tmem14c*), indicating a stimulatory response (Fig. 3A). Further research is needed to clarify these genes' functional roles.

In the cap mesenchyme, analysis revealed 294 genes with significant differential expression. Key genes such as *Edar* and *Fhod3* were upregulated, while *Ptgs2*, *Hspb1*, and *Aif1l* were consistently downregulated across multiple treatment groups (Figs. 3B, 3C, 3D), highlighting their likely involvement in the Wnt signaling response.

The enrichment in the Hippo signaling pathway (Fig. 3E) suggests potential coordination with the Wnt pathway in controlling organ size and promoting cell proliferation and survival [60], [61]. This is consistent with previous report showing crosstalk between Wnt and Hippo signaling [57]. YAP/TAZ activation could promote the differentiation of nephron progenitor cells into interstitial myofibroblastic cells in the kidney [62]. YAP is essential for nephrogenesis, while NF2 and LATS are needed for the morphogenesis of ureter branching [63], [64].

The identification of the focal adhesion pathway (Fig. 3E) underscores the significance of cell-matrix interactions in cap mesenchyme organization and nephron development. Previous report show canonical Wnt signaling induces focal adhesion endocytosis [65]. This study shows for the first time that Wnt/β-catenin could potentially regulate focal adhesion pathway via gene regulation.

Additionally, the enrichment of the AGE-RAGE signaling pathway may link gene expression during development with increased susceptibility to diabetic complications in adulthood (Fig. 3E). Previous study suggests a potential crosstalk between AGE-RAGE signaling and Wnt/β-catenin signaling in adult diabetic rats [66]. Thus, this study presents the first evidence that Wnt/β-catenin could potentially regulate AGE-RAGE signaling in the developing kidney.

GO term analysis highlights the importance of the collagen−containing extracellular matrix, extracellular matrix binding, extracellular matrix structural constituent, SMAD binding, regulation of cell−substrate adhesion. mesenchyme development and mesenchymal cell differentiation in kidney morphogenesis and structural integrity (Fig. 3E). Extracellular matrix is important for kidney development and function [67], [68]. Sustained activation of Wnt/β-catenin accelerates the progression of AKI to CKD, characterized by interstitial myofibroblast activation and excessive extracellular matrix deposition, whereas blockade of Wnt/β-catenin prevents this progression in the adult kidney context [69]. This finding represent the first to show Wnt/β-catenin regulates extracellular matrix in the developing kidney. Previous study shows that phospho-SMAD1 and β-catenin are overexpressed in human fetal dysplastic renal tissue suggesting that dysregulation of these signaling effectors is pathogenic in human renal dysplasia [70]. The current study showing that Wnt/β-catenin regulates SMAD binding further support crosstalk exist between these two signaling.

The trend of decreased expression in DEGs indicates a need for finely-tuned Wnt signaling during development (Supplementary Figure 2). Imbalances in this signaling could potentially lead to developmental abnormalities, underscoring the critical nature of balanced Wnt pathway activity [71].

The study indicates that activation of Wnt signaling in the ureteric bud and cap mesenchyme triggers distinct yet overlapping signaling cascades, reflecting their respective roles in renal development. The ureteric bud forms the collecting system, while the cap mesenchyme generates nephron progenitors. Understanding these specific and common signaling pathways may enhance knowledge of kidney organogenesis and identify therapeutic targets for renal pathologies related to developmental anomalies or diabetes-related complications. Caution is advised when interpreting the results due to the differing methodologies of Wnt activation in the datasets: genetic manipulation *in vivo* (GSE20325) and pharmacological treatment *in vitro* (GSE39583).

The analysis of the β-catenin ChIP-seq and gene expression datasets from this study has provided valuable insights into the transcriptional landscape influenced by β-catenin, a key mediator in the Wnt signaling pathway. By integrating ChIP-seq data with gene expression profiles, the study identified 221 genes as potential direct targets of β-catenin (Fig. 4A, Supplementary Table 6), indicating a broad regulatory role for this transcription factor in the mouse genome.

The identification of 31 genes consistently altered by β-catenin activation in cap mesenchyme across three experimental conditions highlights their potential role in kidney development and suggests a direct regulatory relationship (Figs. 4B, 4C, 4D). This analysis revealed genes such as *Dpp6*, *Tm9sf4*, *Tnfrsf19*, and *Fgf12* as recurrent targets (Figs. 4B, 4C, 4D), suggesting their significance in β-catenin signaling pathways. Notably, Tnfrsf19 competes with Wnt receptors [72], and Tm9sf4 influences the canonical Wnt pathway [73], indicating a feedback mechanism within Wnt signaling. The involvement of Dpp6 points to β-catenin’s influence extending to neurodevelopment, while the consistent appearance of *Fgf12* underscores β-catenin's role in modulating growth factor signaling, critical for diverse developmental processes. It was reported that β-catenin regulates *TNFRSF19* expression in colorectal cancer cells [74]. This study shows for the first time that *Dpp6*, *Tm9sf4*, and *Fgf12* are potential direct targets of Wnt/β-catenin signaling.

Single-cell analysis of the mouse kidney dataset GSE131240 has elucidated cellular diversity and gene expression changes due to Wnt/β-catenin signaling activation. Cross-referencing with the Mouse Cell Atlas revealed 787 DEGs across various cell types in the mouse fetal kidney, influenced by β-catenin. Notably, many of these DEGs are categorized to Macrophage_Apoe high cells during kidney development (Figs. 5B, 5C, Supplementary Table 7). Embryonic kidney macrophages originate from multiple progenitor sources and are present from early stages [4], [5], [75], [76]. Recent single-cell sequencing studies on the human fetal kidney reveal that macrophages are the predominant immune cell type in the developing kidney [3], [4], playing diverse roles such as mediating cell death and clearance, ureteric bud branching morphogenesis, nephron formation, blood and lymphatic vessel development [5]. In the adult kidney, Wnt/β-catenin signaling induces M2 macrophage polarization and contributes to kidney fibrosis [77]. Macrophages are both a source and a target of Wnt signals [78], highlighting their crucial role in the signaling pathway's function during renal development. This study is the first to suggest that Wnt/β-catenin may influence kidney development by modulating macrophage function.

The categorization of DEGs into nineteen renal cell subtypes has provided insight into the specific cellular responses to β-catenin signaling. Differential gene expression patterns in proximal tubule cells, fibroblasts, nephron progenitor cells, and podocytes highlight the broad impact of β-catenin signaling on renal cell lineages. For instance, the downregulation of *Ace2* and *Ppp1r14a* in Fibroblast_Mgp high cells, and the upregulation of genes like *Cpe* and *Mmp11* in Fibroblast_Dlk1 high cells suggest that β-catenin signaling may influence fibroblast cells in the kidney (Fig. 5D). The study also reveals the regulation of genes by Wnt/β-catenin signaling in nephron progenitor cells and other renal cells, including those in the collecting ducts and distal convoluted tubules, pointing to a multifaceted role for this pathway in kidney development.

This single-cell analysis demonstrates that β-catenin activation modulates gene expression across various kidney cell types, highlighting the complexity of Wnt/β-catenin signaling in kidney development and function. These insights establish a foundation for future research to delineate the cell-specific functions of β-catenin and its potential role in kidney diseases. Further empirical investigation is required to clarify the impact of these gene expression changes on kidney cell function and pathology.

This analysis has some limitations. When comparing the Wnt effects on ureteric bud or cap mesenchyme, treating embryonic kidneys with Wnt signaling activator BIO might have broader effects than the gene knockout experiment. However, from the apparent difference of the top-ranked gene list and enriched gene pathways, this analysis at least gives us a glimpse of how Wnt/β-catenin could have different downstream effects during fetal kidney development.

Evidence suggest that Wnt can interact with other major signaling pathways, including bone morphogenetic protein (BMP) signaling, TGF-β signaling, Notch signaling, Hippo/Yap pathway, Hedgehog (Hh) signaling, fibroblast growth factor (FGF) signaling, parathyroid hormone (PTH) signaling [57]. Beyond the Hippo pathway (Figs. 1D, 3E), this study shows that Wnt/β-catenin regulates multiple genes involved in other pathways, pointing to potential interactions. This research lays the groundwork for further exploration of Wnt/β-catenin crosstalk with these signaling pathways.

In conclusion, the current study has identified potential target genes of β-catenin, providing a foundation for further investigation into the role of Wnt/β-catenin signaling in regulating these genes. Wnt/β-catenin regulates a different set of genes in ureteric bud and cap mesenchyme. Wnt/β-catenin regulated genes are constantly highly presented in Macrophage_Apoe high cell as marker genes. Functional studies, such as knockout or overexpression experiments specifically in ureteric bud, cap mesenchyme or Macrophage_Apoe high cell, should be conducted to determine how these gene expression changes impact kidney development and function. Further research is needed to elucidate the precise mechanisms by which β-catenin regulates these genes and to understand the biological significance of these interactions. This could have potential implications for developing therapeutic strategies targeting the Wnt/β-catenin signaling pathway in diseases.

## Funding

This work was supported by the 10.13039/501100003453Natural Science Foundation of Guangdong Province, China (No. 2022A1515012172, 2020A1515011303), the Science, Technology, and Innovation Commission of Shenzhen Municipality (No. JCYJ20190809104207736, JCYJ20220530141616038), Nanshan District Health System Major Science and Technology Project - High-level Hospital Health Science and Technology Special Project - Outstanding Youth Fund Project (NSZD2023029). Huazhong University of Science and Technology Union Shenzhen Hospital Funds (No. YN2022013) and Nanshan District Health System Major Science and Technology Project (No. NSZD2023015).

## Author statement

We the undersigned declare that this manuscript is original, has not been published before and is not currently being considered for publication elsewhere. We confirm that the manuscript has been read and approved by all named authors and that there are no other persons who satisfied the criteria for authorship but are not listed.

## CRediT authorship contribution statement

**Wuping Sun:** Supervision, Resources. **Chang Sun:** Methodology, Investigation. **Yujin Zhou:** Methodology, Investigation. **Quan Liu:** Resources. **Hui Zhao:** Writing – review & editing, Writing – original draft, Visualization, Validation, Software, Methodology, Formal analysis, Data curation. **Dixian Luo:** Resources. **Peide Zhu:** Resources. **Yifan Li:** Writing – review & editing, Validation, Funding acquisition, Data curation, Conceptualization. **Hui Gong:** Resources, Methodology, Investigation, Funding acquisition. **Ailin Qiu:** Methodology, Investigation. **Xiaoxiao Wu:** Supervision, Resources. **Jinde Zhang:** Investigation. **Xiaosha Wen:** Investigation.

## Declaration of Generative AI and AI-assisted technologies in the writing process

During the preparation of this work the author(s) used Chatgpt 4 in order to improve language and readability. After using this tool/service, the author(s) reviewed and edited the content as needed and take(s) full responsibility for the content of the publication.

## Declaration of Competing Interest

All the authors declared no competing interests.

## Data Availability

The datasets generated during the current study are available in the Gene Expression Omnibus (GEO) repository, with accession number GSE131240. The custom code used in the analyses is available in the GitHub repository at https://github.com/zlhlouis/mouse-kidney-paper/tree/main.

## Appendix A Supplementary material

Supplementary material **Supplementary Figure 1** The correlation of the GO Terms and genes for the dataset GSE131240. The figure shows the genes correlated with GO terms, the circle in the middle represents the GO term, and genes are connected by a line segment.

.

Supplementary material **Supplementary Figure 2** The GO bubble plot for the 294 significantly differentially expressed genes in cap mesenchyme (dataset GSE39583). The x-axis was the z-score. The positive value represents the GO term, which is more likely to be increased, while the negative represents the GO term, which is likely to be decreased.

.

Supplementary material **Supplementary Figure 3** The experimental design involved treating cap mesenchyme cells, which are undifferentiated nephron progenitors, with 4 μM BIO to activate Wnt signaling, as documented in dataset GSE39583.
