# Supplementary figures and images for: Deciphering the cellular and molecular landscapes of Wnt/β-catenin signaling in mouse embryonic kidney development
Source: Comput Struct Biotechnol J. 2024 Sep 2;23:3368–78. doi: 10.1016/j.csbj.2024.08.025 (PMC11416353; doi:10.1016/j.csbj.2024.08.025)

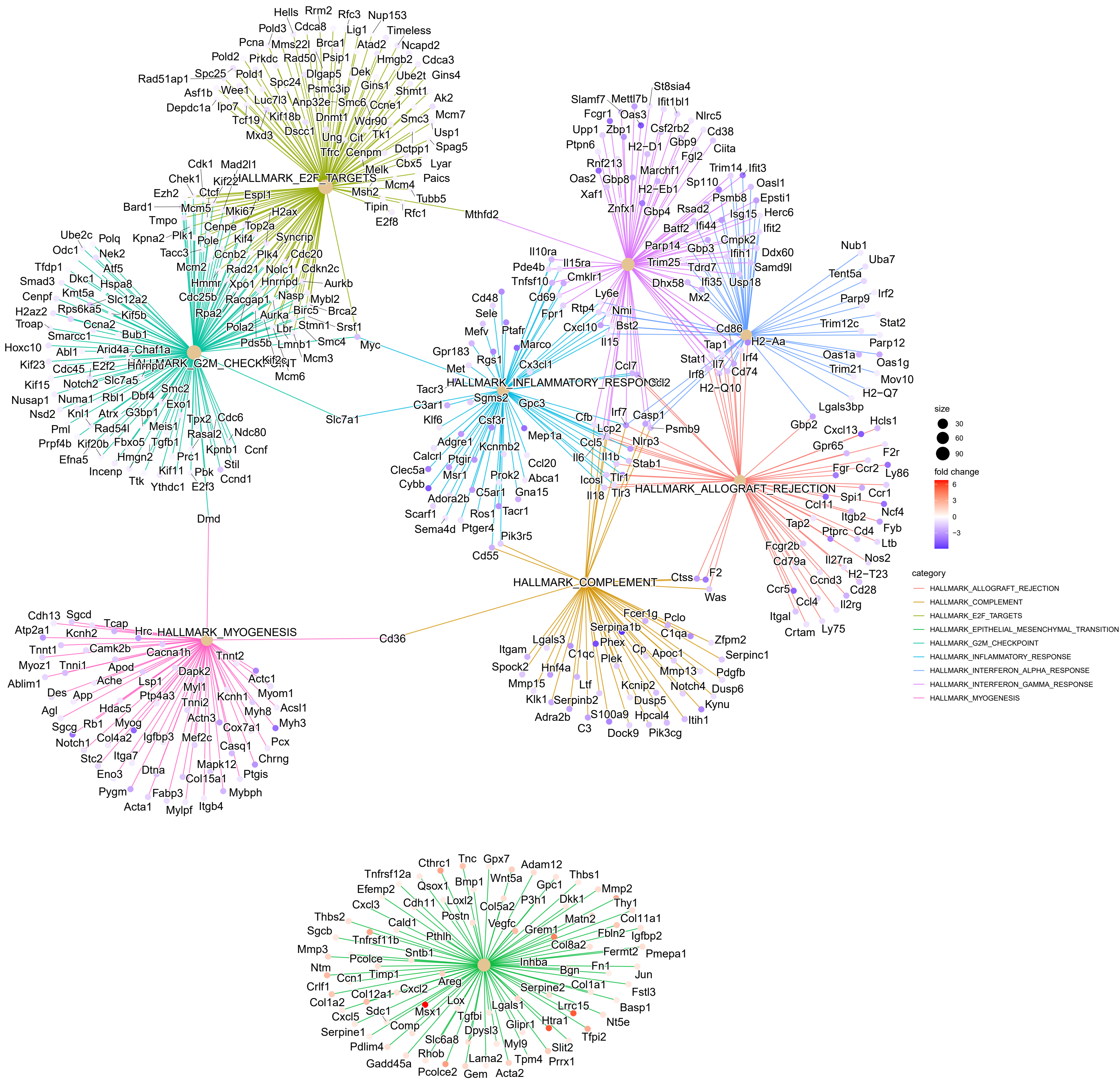

Supplement: Supplementary file 1 — Supplementary material Supplementary Figure 1 The correlation of the GO Terms and genes for the dataset GSE131240. The figure shows the genes correlated with GO terms, the circle in the middle represents the GO term, and genes are connected by a line segment. [file mmc1.pdf]

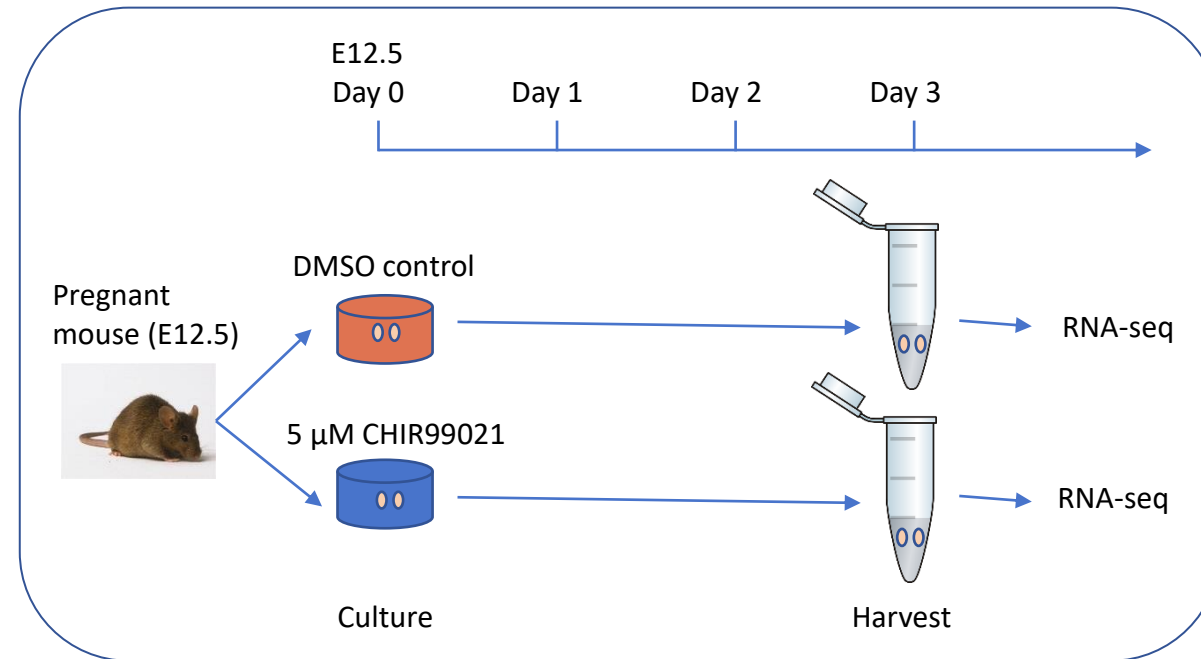

Supplement: Supplementary file 4 — Supplementary material [file mmc4.pdf]
