# Supplementary material for: Deciphering the cellular and molecular landscapes of Wnt/β-catenin signaling in mouse embryonic kidney development
Source: Comput Struct Biotechnol J. 2024 Sep 2;23:3368–78. doi: 10.1016/j.csbj.2024.08.025 (PMC11416353; doi:10.1016/j.csbj.2024.08.025)
Supplement: Supplementary file 2 — Supplementary material Supplementary Figure 2 The GO bubble plot for the 294 significantly differentially expressed genes in cap mesenchyme (dataset GSE39583). The x-axis was the z-score. The positive value represents the GO term, which is more likely to be increased, while the negative represents the GO term, which is likely to be decreased. [file mmc2.pdf]

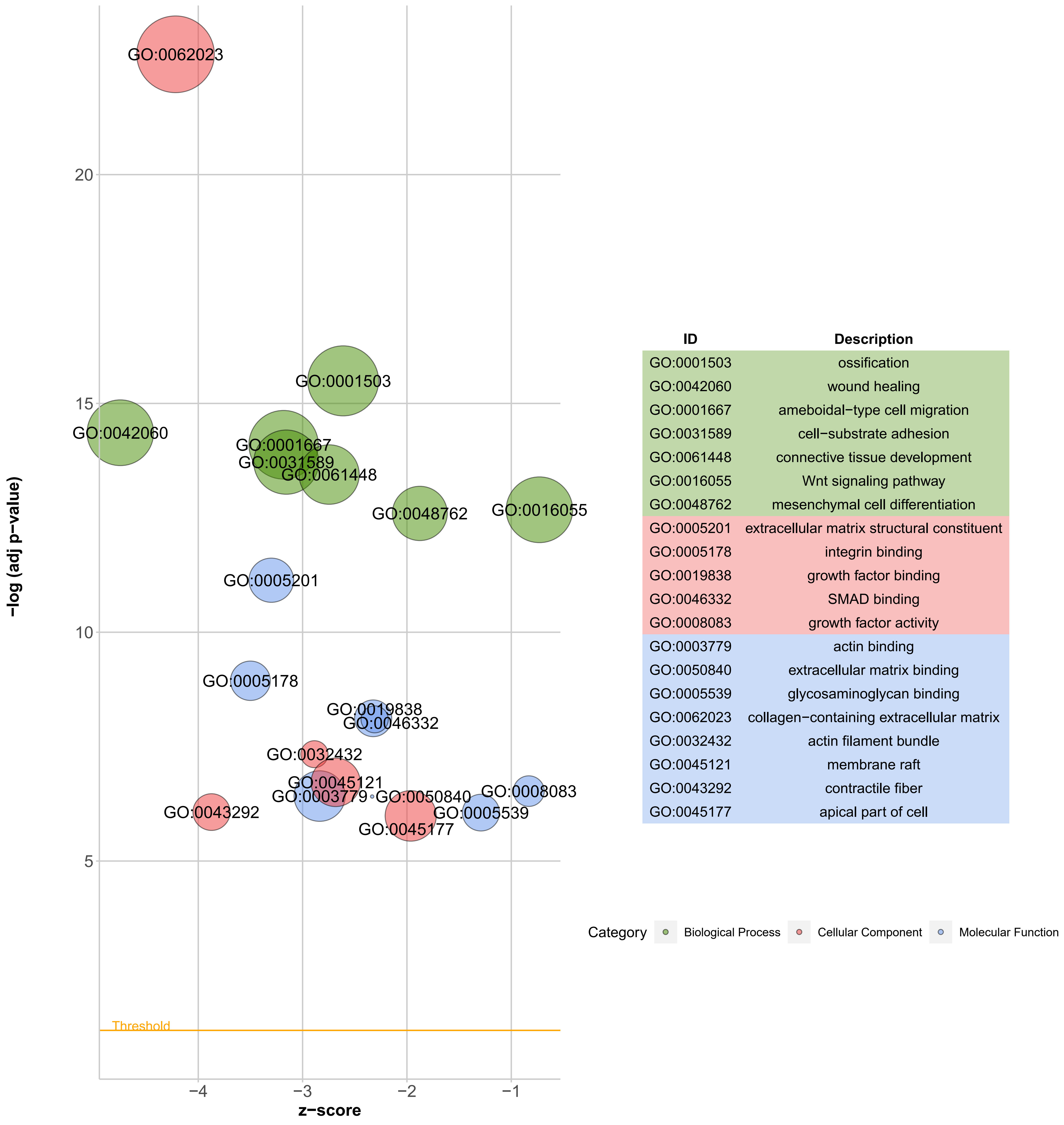
GO:0062023
GO:0001503
GO:0042060
GO:0001667
GO:0031589
GO:0061448
GO:0048762
GO:0016055
GO:0005201
GO:0005178
GO:0019838
GO:0046332
GO:0032432
GO:0045121
GO:0003779
GO:0050840
GO:0008083
GO:0043292
GO:0005539
GO:0045177
Threshold
20
15
10
5
−4
−3
−2
−1
−log (adj p−value)
z−score
ID Description
GO:0001503 ossification
GO:0042060 wound healing
GO:0001667 ameboidal−type cell migration
GO:0031589 cell−substrate adhesion
GO:0061448 connective tissue development
GO:0016055 Wnt signaling pathway
GO:0048762 mesenchymal cell differentiation
GO:0005201 extracellular matrix structural constituent
GO:0005178 integrin binding
GO:0019838 growth factor binding
GO:0046332 SMAD binding
GO:0008083 growth factor activity
GO:0003779 actin binding
GO:0050840 extracellular matrix binding
GO:0005539 glycosaminoglycan binding
GO:0062023 collagen−containing extracellular matrix
GO:0032432 actin filament bundle
GO:0045121 membrane raft
GO:0043292 contractile fiber
GO:0045177 apical part of cell
Category
Biological Process
Cellular Component
Molecular Function
