# Supplementary material for: Deciphering the cellular and molecular landscapes of Wnt/β-catenin signaling in mouse embryonic kidney development
Source: Comput Struct Biotechnol J. 2024 Sep 2;23:3368–78. doi: 10.1016/j.csbj.2024.08.025 (PMC11416353; doi:10.1016/j.csbj.2024.08.025)
Supplement: Supplementary file 3 — Supplementary material Supplementary Figure 3 The experimental design involved treating cap mesenchyme cells, which are undifferentiated nephron progenitors, with 4 μM BIO to activate Wnt signaling, as documented in dataset GSE39583. [file mmc3.pdf]

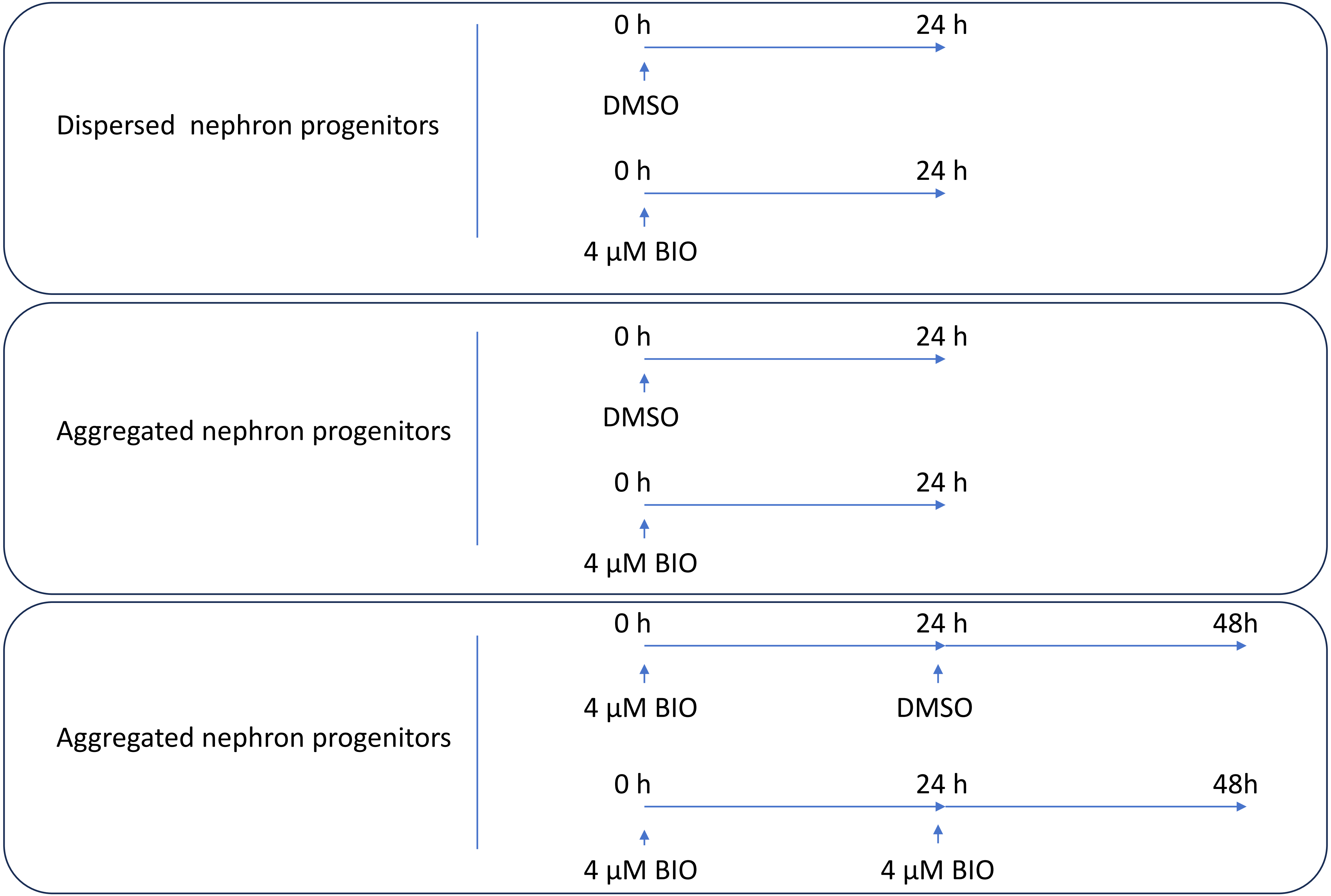
Dispersed nephron progenitors
0 h
24 h
DMSO
0 h
24 h
4 µM BIO
Aggregated nephron progenitors
0 h
24 h
DMSO
0 h
24 h
4 µM BIO
Aggregated nephron progenitors
0 h
24 h
48h
4 µM BIO
DMSO
0 h
24 h
48h
4 µM BIO
4 µM BIO
